# News and Events

**Published:** 2009

**Authors:** T. Ganesh

**Affiliations:** Department of Radiation Oncology, King Fahad Specialist Hospital, Dammam, Kingdom of Saudi Arabia. Email: sptgnadar@yahoo.com

## Mortality and Cancer Risks for Radiation Workers

According to a study by the Health Protection Agency, published in the British Journal of Cancer (Volume 100, Issue 1, pages 206 to 218), the risk of developing cancer, among radiation workers, increases with the dose of ionizing radiation they are exposed to. The study is said to be in continuation of an earlier study started in 1976. It claims to provide the most precise estimates, to date, of mortality and cancer risks following occupational radiation exposure, and strengthens the scientific evidence for a rise in risks from these exposures. The cancer risk estimates are consistent with the international radiation protection standards, both for leukemia and all other cancers combined.

The study also shows that the overall mortality in UK's 175,000 radiation workers is lower than that in the general population. It concludes that the radiation workers have lower cancer risks than the general population. This “healthy worker effect” has been observed in studies of many other occupational groups.

From: http://www.hpa.org.uk/webw/HPAweb&HPAwebStandard/HPAweb_C/1231252392462?p=1231252394302

## Image Gently Campaign Gains Momentum

The *Image Gently* campaign, launched a year ago, is fast gaining momentum with more than 1500 professionals so far taking the pledge to image gently. The message will be given priority in the staff communication this year and the focus will be on computed tomography (CT) scans. To help communication with parents, the Image Gently Alliance has developed two downloadable brochures titled “What Parents Should Know about CT scans for Children” and “What Parents Should Know about Medical Radiation Safety”.

The campaign seems to have started yielding the desired results. As reported by two of the largest academic children's hospitals in USA, the volume of CT procedures performed by the hospitals has declined in the past two years, even as overall imaging volumes have steadily increased.

The brochures can be downloaded from: *http://www.pedrad.org/displaycommon.cfm?an=1andsubarticlenbr=388*

From: www.imagegently.org, www.aapm.org and www.auntminnie.com dated Dec 12, 2008

## Nuclear Regulatory Commission deploys National Source Tracking System

The Nuclear Regulatory Commission, USA, has deployed its National Source Tracking System (NSTS), a centralized national registry, to provide cradle-to-grave accounting of certain high-risk radioactive materials used in industry, medicine, and research. The system, besides monitoring the location and use and disposal of certain radiation sources, will also improve the ability of regulators to detect and act upon inventory discrepancies, respond to emergencies, and verify the legitimate import, export, ownership, and use of sources. What makes the system different from other such systems in practice worldwide is that all the licensees will report to the NSTS primarily, over the Internet, using a secure, authenticated link. Licensees will have access to the information on their facility, but will not have access to information about other licensees. Members of the public will not have access to the data.

From: http://www.nrc.gov/reading-rm/doc-collections/news/2009/09-002.html

## Independent Review of Radiotherapy Under-Dose of Several Hundred Patients in Australia

During the period between July 2004 and July 2006, patients treated in a linear accelerator in an Australian center, received a dose 5% lower than their prescribed doses. The error occurred due to incorrect measurement of the ‘beam energy ratio’ for the 6 MV beam in July 2004 and feeding of this data into the treatment planning system (TPS). The TPS used this ‘beam energy ratio’ for the calculation of monitoring units, which resulted in an under-dosing of up to 5% in a dose. In July 2006 the error was noticed and corrected immediately.

An independent review to look into the event is now available for reading. Professor Geoff Delaney, who led the review panel, concluded that the under-dose would not have an impact on the vast majority of cancer patients. The report, however, indicates that there may be a small number of patients in three different tumor groups who may have suffered a clinical impact.

From: http://www.health.sa.gov.au/Default.aspx?tabid=555 and http://rpop.iaea.org/RPOP/RPoP/Content/News/review-radiotherapy-Australia.htm

## Report on a Serious Adverse Event Leading to Significant Overdose Recently Made Public

The *International Atomic Energy Agency* (IAEA) website on Radiation Protection of Patients has made available a full report of a Review Panel constituted to look into a serious radiotherapy incident which occurred few years back. The place and dates are not disclosed in the report submitted in May 2005. The Review Panel concluded that the serious adverse incident was primarily due to inadvertent human error. While the treatment plan parameters from the TPS were being manually entered into the linac computer's database, a wedged field was inadvertently entered as an open field. However, the monitoring unit value remained the same as for the wedged field. This led to the patient receiving 2.5 times the intended dose. The patient was being treated for the overdose she received at the time of the report.

The incident underlines the importance of transferring treatment plan data through the networking of TPS with a linac computer and highlights the high risks associated with manual entry of the treatment plan parameters.

From: http://rpop.iaea.org/RPOP/RPoP/Content/News/radiotherapyincident.htm

## World's First Hybrid Magnetic Resonance Imaging (MRI) - Linac Unit

Medical Physicists from Cross Cancer Institute (CCI), Edmonton Alberta, Canada produced the first image from a linac-magnetic resonance (MR) hybrid system on December 10, 2008. The MR images obtained during 6MV irradiation (when the beam was ON) did not show significant distortions and were very similar to those obtained prior to irradiation. However there was a small difference in the signal-to-noise ratio between the two sets of images. The linac-MR hybrid system consisted of a 6 MV linac, mounted on the open end of a biplanar, low-strength (0.2T) MRI magnet. Both the linac and the MR system were mounted on a single gantry that would rotate around the patient. CCI Researchers are investigating the influence of this scheme, dubbed as rotating-biplanar geometry, on dose distributions.

From: http://www.aapm.org/pubs/enews/documents/Fallone_info.doc and www.linac-mr.ca

## Recent Publications of Interest from the International Atomic Energy Agency

### Quality Management Audits in Nuclear Medicine Practices

Quality management (QM) is essential for modern nuclear medicine departments. This publication presents a method for conducting a systematic annual audit process. The key outcome of the QM methodology should be a culture of reviewing essential elements of the clinical service. The QM methodology described in this publication is designed to be applied to a variety of economic circumstances and will be of use to those involved in the development or implementation of QM systems.

http://www-pub.iaea.org/MTCD/publications/PDF/Pub1371_web.pdf

### Radiation Protection In Newer Medical Imaging Techniques: PET/CT

Safety Reports Series No. 58

This Safety Report reviews radiation protection issues arising from the use of Positron Emission Tomography – Computed Tomography (PET/CT) and offers guidance on dose management and optimization. It provides data on patient dose and risk levels, as well as, information for practitioners, on optimizing techniques.

http://www-pub.iaea.org/MTCD/publications/PDF/pub1343_web.pdf

### A Guide to Clinical Pet in Oncology: Improving Clinical Management of Cancer Patients

IAEA TECDOC Series No. 1605

This publication is intended to assist health managers, nuclear medicine physicians, and referring physicians involved in the management of cancer patients through further application of PET technology. This publication aims to provide Member States with a guide and a useful resource for specialists in nuclear medicine and in oncology, to improve the clinical management of cancer patients through these techniques.

http://www-pub.iaea.org/MTCD/publications/PDF/te_1605_web.pdf

### The Role of PET/CT in Radiation Treatment Planning for Cancer Patient Treatment

IAEA TECDOC Series No. 1603

This publication is a resource for specialists in nuclear medicine and radiation oncology on the coupling of positron emission tomography and X ray computed tomography (PET/CT), to more accurately identify cancer cells within the body. The joint use of PET and CT provides superior imaging of the existing disease, and therefore, promises to minimize adverse effects of radiation therapy. By marking the presence of cancerous cells more precisely, it facilitates more precise irradiation and also enables an increase in the dose of radiation therapy delivered to the malignant cells. This leads to better tumor control and improved survival of patients.

http://www-pub.iaea.org/MTCD/publications/PDF/te_1603_web.pdf

### Operational Guidance on Hospital Radiopharmacy: A Safe and Effective Approach

Clinically safe, effective, and economic practices in the area of hospital radiopharmacy can strengthen the overall performance of nuclear medicine services. This guidance provides practical points at different levels of operation including staff training, facilities, radiopharmaceutical practices, record keeping, and quality control. Therefore, it is an essential read for nuclear medicine physicians, radiologists, and radiopharmacists, who take responsibility to ensure concordance with internationally recognized practices.

http://www-pub.iaea.org/MTCD/publications/PDF/Pub1342_web.pdf

### Report of IAEA Meeting on Radiation Safety Impact of Newer Technologies

In October 2008, IAEA organized a meeting on “*Radiation safety impact of newer imaging and radiation therapy technologies in medicine”* in Buenos Aires, Argentina.

The report of the meeting raises concerns on the marked increase in both the dose per person and the collective dose due to medical radiological procedures carried out every year globally, and states that the increase in exposure is not bad so long as clinical benefits outweigh the risks, and risks have been minimized to maintain the benefits.

Salient points highlighted in the report are: (i) Patient doses in radiography have decreased significantly over the last few decades, whereas, in CT they have registered an increase; (ii) Radiotherapy is becoming more complex and manual calculations are not feasible; (iii) A very large part of the unnecessary exposure stems from unjustified examination; (iv) The speed at which technology is changing poses challenges to the development, assessment, and implementation of radiation dose management programs; (v) Requiring audit may be more effective than strict regulatory controls; (vi) Develop a methodology for long-term record of patient doses.

http://rpop.iaea.org/RPOP/RPoP/Content/Documents/Whitepapers/TMIRPAReport.doc

## Calendar of Events

### International

1. **Annual Scientific Meeting and CCPM Symposium**
  Place:Victoria, BC, Canada
  Meeting Dates: July 21 to 24, 2009
  Last date for submission of abstract: To be announced For more details, log onto: http://members.shaw.ca/COMP2009/
2. **American Association of Physicists in Medicine - 51^st^ Annual Meeting**
  Place: Anaheim, California, USA
  Meeting Dates: July 26 to 30, 2009
  Last date for submission of abstract: March 4, 2009
  For more details, log onto: http://aapm.org/meetings/09AM/
3. **World Congress on Medical Physics and Biomedical Engineering – 11^th^ International Congress of the IUPESM**
  Place: Munich, Germany
  Meeting Dates: September 7 to 12, 2009
  Last date for submission of abstract: February 22, 2009
  For more details, log onto: http://www.wc2009.org/WORLD-CONGRESS-2009/Pages/Home.aspx
4. **American Society for Radiation Oncology - 51^st^ Annual Meeting**
  Place: Chicago, Illinois, USA
  Meeting Dates: November 1 to 5, 2009
  Last date for submission of abstract: April 2, 2009
  For more details, log onto: http://astro.org/Meetings/AnnualMeetings/CallForAbstracts/

